# Differential effects of hormone therapy formulations on Parkinson’s disease risk: a systematic review and meta-analysis

**DOI:** 10.1007/s10072-026-09243-6

**Published:** 2026-07-22

**Authors:** Victor Fellipe Bispo Macedo, Alana Madeiro de Melo Barboza

**Affiliations:** 1 Neurology Service of Hospital Santa Casa de Misericórdia de Maceió, Rua Barão de Maceió, 346 - Centro, Maceió, 57020-360 AL Brazil; 2 Universidade de Maceió, UNIMA, Maceió, Alagoas Brazil; 3 Centro Universitário CESMAC, Maceió, Alagoas Brazil

**Keywords:** Parkinson disease, Hormone replacement therapy, Menopause, Estrogens, Progestins

## Abstract

**Background:**

The relationship between menopausal hormone therapy (HT) and Parkinson’s disease (PD) risk remains controversial, with inconsistent findings potentially driven by differences in hormonal formulations.

**Methods:**

We conducted a systematic review and meta-analysis following PRISMA 2020 guidelines. MEDLINE/PubMed and EMBASE were searched between January 8 and March 14, 2026. Observational studies evaluating the association between menopausal HT and PD risk were included. Effect estimates were pooled using random-effects models. Subgroup analyses were performed according to HT formulation (estrogen-only vs. combined estrogen–progestin therapy). A multilevel meta-analysis was conducted to account for within-study dependence.

**Results:**

Fourteen studies including 753,749 participants (4,433 PD cases) were analyzed. Overall, HT was not significantly associated with PD risk (RR 1.08; 95% CI 0.94–1.23; I² = 49.4%). In subgroup analyses, combined therapy was associated with an increased PD risk (RR 1.40; 95% CI 1.07–1.82), whereas estrogen-only therapy showed no significant association (RR 1.01; 95% CI 0.81–1.27). Multilevel analysis yielded consistent results (combined: RR 1.33; 95% CI 1.00–1.77; estrogen-only: RR 1.03; 95% CI 0.83–1.27), with no statistically significant interaction between formulations (*p* = 0.12).

**Conclusions:**

Combined menopausal HT was associated with an increased risk of PD, while estrogen-only therapy showed no significant association. Although differences between formulations were not statistically significant, these findings suggest that hormone composition may influence neurological outcomes and warrant further investigation into individualized HT strategies.

**Supplementary Information:**

The online version contains supplementary material available at 10.1007/s10072-026-09243-6.

## Highlights

Menopausal hormone therapy was not associated with overall Parkinson’s disease risk.

Combined estrogen–progestin therapy was associated with increased PD risk.

Estrogen-only therapy showed no significant association with PD.

Multilevel meta-analysis confirmed results accounting for within-study dependence.

No statistically significant interaction was observed between hormone formulations.

**Supplementary Information:**

The online version contains supplementary material available at 10.1007/s10072-026-09243-6.

## Background

Parkinson’s disease (PD) is the second most common neurodegenerative disorder worldwide and represents one of the leading causes of neurodegenerative disorders [1–4]. It is characterized by the progressive loss of dopaminergic neurons in the substantia nigra, resulting in motor and non-motor manifestations [5].

Epidemiological studies consistently demonstrate that PD incidence is approximately twofold higher in men than in women. In addition, women tend to develop PD predominantly after menopause, suggesting a potential protective role of endogenous sex hormones, particularly estrogens [6–8]. This hypothesis is supported by observational data linking PD risk to reproductive factors associated with reduced lifetime estrogen exposure, including shorter fertile lifespan, higher cumulative duration of pregnancy and earlier menopause [6].

Furthermore, among women with PD, shorter reproductive lifespan has been associated with greater disease severity, reflected by higher Hoehn and Yahr stages and increased levodopa equivalent daily dose (LEDD) requirements [7]. These findings further support the concept of a neuroprotective role of estrogens.

Experimental and clinical evidence suggests that estrogens may modulate neuronal survival and function, synaptic plasticity, and dopaminergic neurotransmission within the substantia nigra and striatum [9]. However, the effects of estrogen exposure appear to depend on timing, duration, and physiological context across a woman’s reproductive lifespan [6].

In the postmenopausal setting, hormone therapy (HT) has been investigated as a potential modifier of PD risk. However, findings remain inconsistent, with some studies suggesting an increased risk and others reporting neutral or even protective effects [10]. This inconsistency suggests that the relationship between HT and PD may not be uniform.

One possible explanation for these conflicting results is the existence of a critical window of opportunity for therapeutic benefit for HT [10, 11] and that the effect of HT may depend on its formulation, particularly the use of estrogen alone versus combined estrogen–progestin therapy. Differences in biological effects between these formulations, including potential modulation of dopaminergic pathways, may contribute to the observed heterogeneity.

Therefore, the present study aimed to systematically evaluate the association between HT and the risk of PD, with a specific focus on differences according to hormonal formulation.

## Methods

The review was conducted according to the PRISMA 2020 statement [12] and was prospectively registered in PROSPERO (CRD420261363040). Ethical approval was not required for this systematic review and meta-analysis.

### Search strategy and study selection

A comprehensive search was performed in MEDLINE/PubMed and EMBASE between January 8 and March 14, 2026. The strategy combined controlled vocabulary and keywords related to PD, estrogen, HT and menopause. The full search strategy for each database is provided in Supplementary File 1. In addition, the reference lists of included studies were manually screened to identify potentially eligible articles not captured in the electronic search.

All retrieved records were imported into Rayyan^®^ to facilitate duplicate detection and screening [13]. Two reviewers (VM, AB) independently evaluated titles and abstracts. Full-text articles were retrieved for studies meeting inclusion criteria or when eligibility was uncertain. Disagreements were resolved by consensus.

### Inclusion and exclusion criteria

Studies were included if they: (1) were original research articles; (2) evaluated estrogen exposure (endogenous or exogenous); (3) reported PD as an outcome; and (4) provided effect estimates (hazard ratios, risk ratios, or odds ratios) with corresponding 95% confidence intervals.

Studies were excluded if they lacked extractable data, focused exclusively on disease severity or progression, involved overlapping populations or were not published in English, Spanish or Portuguese. Final eligibility was determined by consensus.

### Data extraction and organization

Data extraction was performed by one investigator (VM) using a standardized data extraction form and independently verified by a second reviewer (AB). Extracted data included: study characteristics and design, population characteristics, diagnostic criteria for PD, exposure characteristics, reproductive variables (when available) and the most adjusted effect estimates with corresponding confidence intervals.

For the primary meta-analysis, exposure was defined as menopausal HT. HT was further classified according to formulation into estrogen-only therapy and combined estrogen–progestin therapy.

Studies reporting only duration-specific estimates, without an overall comparison between HT use and non-use, were excluded from the primary meta-analysis to preserve comparability across studies.

### Quality assessment and risk of bias

Risk of bias was assessed independently by two reviewers using the Newcastle-Ottawa Scale (NOS) [14]. The NOS rates studies across three domains: selection, comparability and exposure. A total score of at least 8 indicates a high quality, between 5 and 6 moderate quality and below 5 low quality. Disagreements were resolved by consensus.

### Statistical analysis

All effect estimates were transformed into their natural logarithm, and standard errors (SE) were calculated from reported 95% confidence intervals. This transformation was performed to normalize the distribution of effect estimates, stabilize variances and allow appropriate weighting in the meta-analysis.

Statistical analyses were performed using R software (version 4.2) with the metafor package. A random-effects model was applied to account for between-study heterogeneity arising from differences in study design, population characteristics, and definitions of hormone exposure. Statistical heterogeneity was assessed using I² statistic, Cochran’s Q test and tau². Given the low incidence of PD, odds ratios and hazard ratios were considered approximations of relative risks.

When studies reported multiple exposure categories, only one effect estimate per study was included in the primary meta-analysis to avoid duplication, ideally the overall one. Because some studies reported both estrogen-only and combined therapy estimates, subgroup-specific meta-analyses were conducted separately. A multilevel meta-analysis model was additionally performed to account for within-study dependence when multiple effect estimates were available.

Publication bias was assessed through visual inspection of funnel plots. Formal statistical tests for funnel plot asymmetry were not performed due to the limited number of included studies.

Sensitivity analyses were conducted using a leave-one-out approach to evaluate the influence of individual studies on the pooled effect estimate.

## Results

### Study selection

**Fig. 1:**
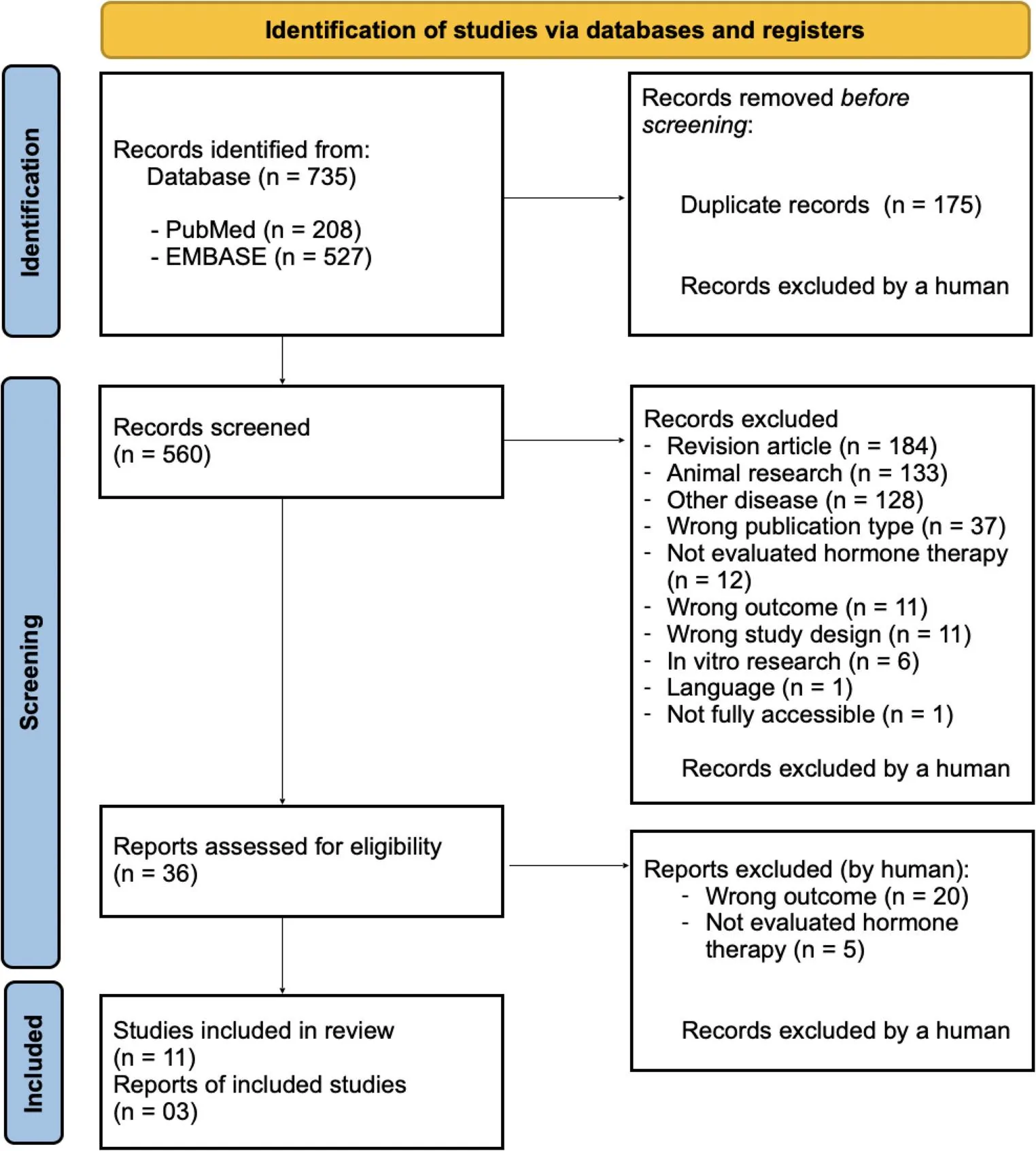
PRISMA flow diagram of study selection

A total of 735 studies were identified through database searches: 208 from PubMed and 527 from EMBASE. After removing 175 duplicates, 560 articles remained for screening. Of these, 11 studies were eligible for inclusion in the meta-analysis and 03 additional studies were identified through manual screening of references lists (Fig. 1). 

### Summary of included studies

These 14 studies included 753,749 participants, of whom 4,433 belonged to the PD group. They were conducted across the United States of America, Denmark, South Korea Italy and France. Diagnostic criteria for PD were heterogeneous: 02 studies used the international classification of disease (ICD) criteria [15], 01 used exclusively the Gelb criteria [16], 01 used the Gelb and UK brain bank criteria [17], 01 used the treating neurologist judgment, 01 not informed and 08 studies relied on expert clinical judgment without mentioning formal criteria (detailed in Supplementary file 2). Follow-up durations ranged from 4 years to 22 years in the cohort studies. The main characteristics of included studies are summarized in Table 1. Based on NOS, 03 studies were moderate risk bias and the others studies were low risk (detailed in Supplementary file 3).

**Table 1:** Summary of included studies

Study (year)	Design	Country	Participants		Follow-up time (years)	Age (SD)	
			PD	Controls		PD	Controls
Marder (1998) [18]	Cohort	USA	87	989	4	71,2 (9.9)	79 (7)
Benedetti (2001) [19]	Case control	USA	72	72	NA	NI	NI
Ascherio (2003) [20]	Cohort	USA	154	77559	18	NI	NI
Currie (2004) [21]	Case control	USA	68	72	NA	71 (8)	66 (8)
Ragonese (2004) [6]	Case control	Italy	131	131	NA	NI	NI
Popat (2005) [22]	Case control	USA	178	189	NA	71.3 (8.7)	70.7 (9.3)
Simon (2009) [23]	Cohort	USA	244	121457	22	NI	NI
Nicoletti (2011) [24]	Case control	Italy	200	299	NA	68.0 (9.5)	61.8 (9.9)
Rugbjerg (2013) [25]	Cohort	Denmark	77	27389	13,3	NI	NI
Liu (2014) [26]	Cohort	USA	410	118759	11	63.5 (5)	61.6 (5.2)
Gatto (2014) [27]	Case control	USA	230	3349	NA	64.1 (9.1)	60.9 (11.4)
Greene (2014) [28]	Case control	Denmark	743	765	NA	NI	NI
Yuk (2023) [29]	Cohort	South Korea	674	302586	7.9	NI	NI
Pesce (2023) [30]	Cohort	France	1165	95700	22	52.6 (6.5)	49.3 (6.6)

*UK* United Kingdom, *ICD* International classification of disease, *PD* Parkinson disease, *SD* Standard deviation, *USA* United States of America, *NA* Not applicable, *NI* Not informed

### Association between menopausal HT and PD

**Fig. 2:**
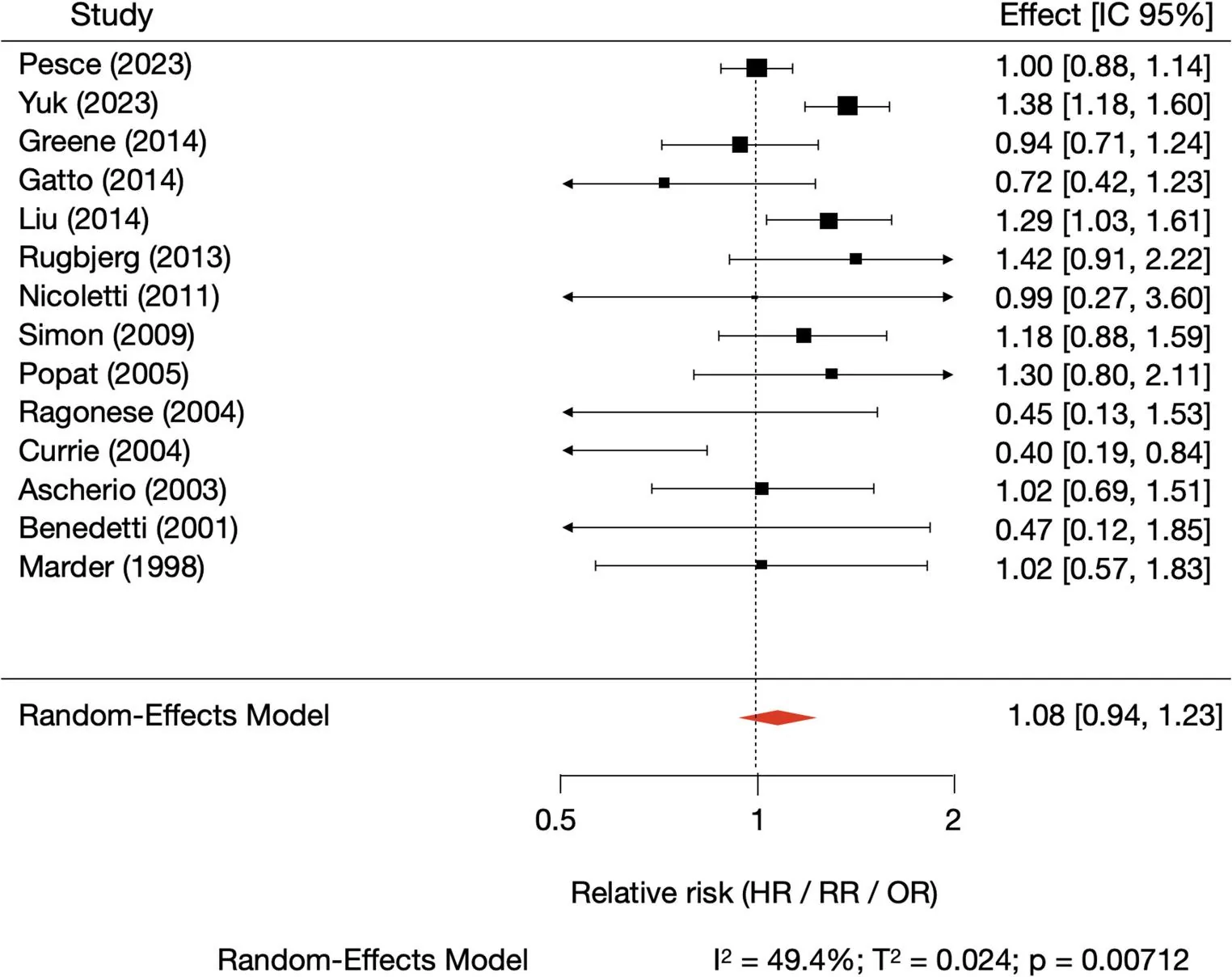
Forest plot of the association between menopausal hormone therapy and risk of PD

The association between HT in PD risk in these fourteen included studies ranged from a protective effect of 0.4 (0.190–0.840) to an increased risk of 1.885 (1.218–2.918). The pooled overall PD risk was 1.08 (95% CI: 0.94–1.23) (Fig. 2), indicating a non-significant association. Heterogeneity was substantial (I² = 49.4%; τ² = 0.024; Q test *p* < 0.0071). Sensitivity analyses indicat ed that no single study had a disproportionate influence on the pooled estimate and funnel plot did not suggest publication bias (detailed in Supplementary file 4). 

**Fig. 3:**
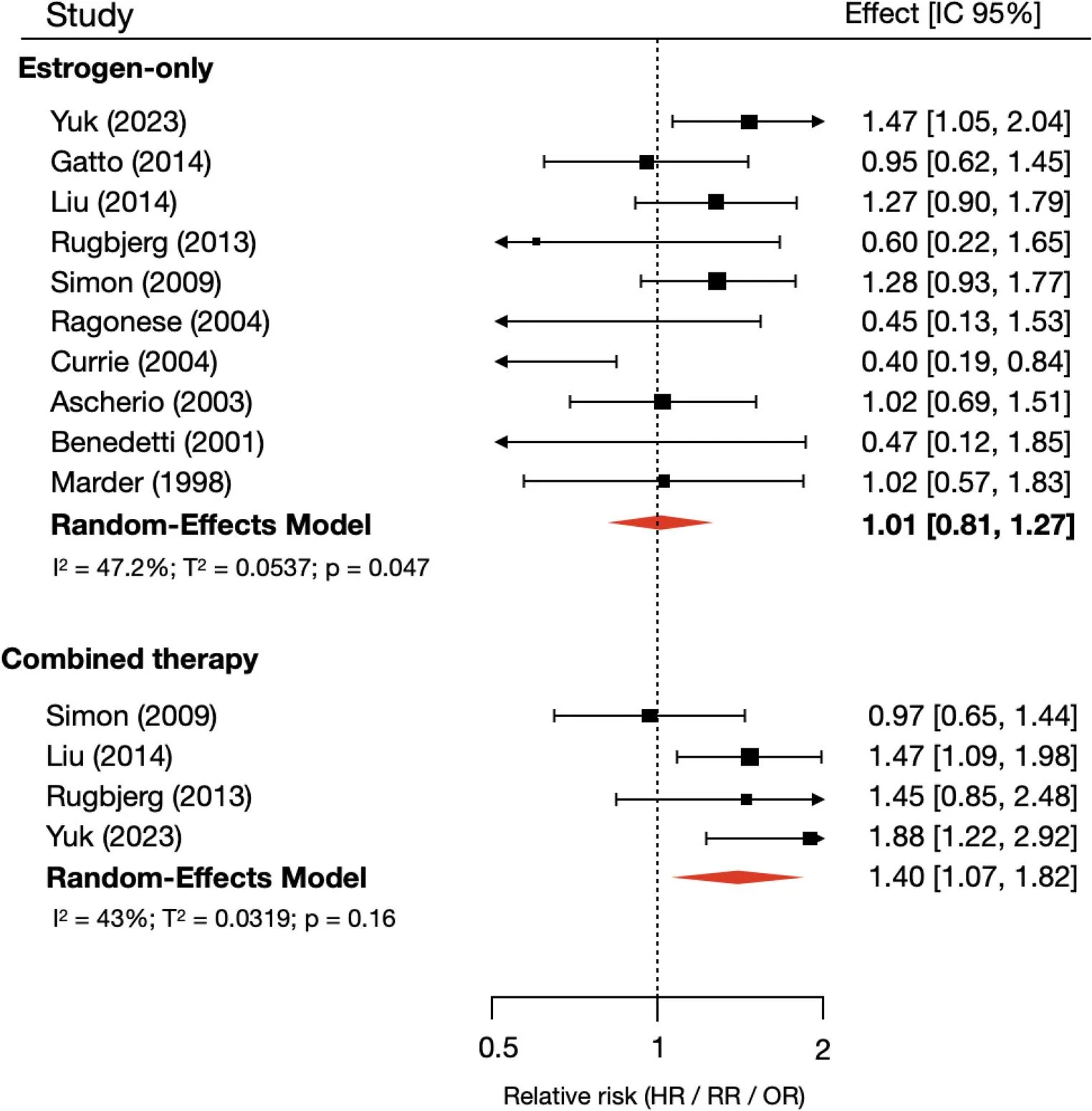
Forest plots of estrogen-only and combined hormone therapy and risk of PD

In HT type subgroup analysis, combined therapy was associated with an increased risk of PD (effect = 1.40; 95% CI 1.07–1.82), whereas estrogen-only therapy was not associated (effect = 1.01; 95% CI 0.81–1.27) (Fig. 3). 

In multilevel meta-analysis accounting for within-study dependence, combined therapy remained associated with an increased risk of PD (RR 1.33; 95% CI 1.00–1.77), whereas estrogen-only therapy showed no significant association (RR 1.03; 95% CI 0.83–1.27). Although a lower effect was observed for estrogen-only therapy compared with combined therapy, the interaction between hormone formulation and PD risk was not statistically significant (*p* = 0.12). However, this lack of statistical significance may reflect limited statistical power rather than a true absence of effect modification. Results from multilevel analysis were consistent with primary subgroup analyses.

## Discussion

This meta-analysis of fourteen observational studies, encompassing diverse populations from USA, Europe, and Asia, indicates that menopausal combined HT is significantly associated with an increased risk of PD (pooled estimate 1.40), which remained consistent in multilevel analysis (RR 1.33). In contrast, no significant associations were observed for menopausal estrogen-only therapy.

Although subgroup analyses suggested a differential pattern according to hormone formulation, this difference was non-significant in multilevel analysis, indicating that results should be interpreted cautiously.

Sex steroids, such as estrogen and progesterone, can be synthesized de novo in the brain by neurons and glial cells through a process known as neurosteroidogenesis [31–33]. These molecules play important roles in neurodevelopment and neuroprotection, and are involved in the regulation and modulation of neurotransmitter systems and neuronal excitability [34].

Estrogen acts in the brain through two main pathways, classical and non-classical signaling pathways. In both, their primary function is to regulate gene transcription in nuclear and mitochondrial DNA via activation of estrogen receptors, particularly ERα and ERβ [34]. Through these mechanisms, estrogen exerts neuroprotective effects by activating anti-apoptotic and cell survival pathways, regulating cellular bioenergetics through increased glucose availability and mitochondrial ATP production, and promoting neurogenesis via the proliferation of neural progenitor cells [34].

Progesterone also acts in the brain through classical and non-classical pathways, ultimately regulating gene transcription via activation of progesterone receptors, particularly PRα and PRβ. Similar to estrogen, progesterone exerts neuroprotective effects by enhancing anti-apoptotic mechanisms, regulating cellular bioenergetics, and promoting neurogenesis [34].

In addition, progesterone plays an important role in glial cell regulation, particularly by stimulating oligodendrocyte formation through the proliferation of oligodendrocyte progenitor cells and the transcription of key components involved in myelin synthesis [34]. It also increases the expression of anti-apoptotic proteins, downregulates pro-apoptotic gene expression, restores mitochondrial function, and modulates inflammatory responses [35]. These effects are particularly relevant in the substantia nigra, a region with some of the highest concentrations of progesterone in the brain [31, 33].

As life expectancy increases, a growing number of women spend a substantial portion of their lives in the postmenopausal state, potentially increasing susceptibility to neurodegenerative diseases due to the loss of steroid-related neuroprotection [35, 36]. To alleviate symptoms and prevent conditions associated with declining hormonal levels, HT has been widely implemented [34]. HT can be administered either as combined therapy (estrogen plus progesterone) or as estrogen-only therapy.

However, the effects of HT on neuroprotection remain inconsistent across studies. Some evidence suggests that estrogen-containing formulations may reduce the risk of neurodegenerative diseases [10]. In contrast, other studies have shown that when initiated in the late postmenopausal period, HT does not improve neuronal function, particularly cognitive outcomes, and may even exert adverse effects [37].

In PD, several studies have demonstrated alterations in the profile of neuroactive steroids in both the central nervous system and peripheral tissues. Cerebrospinal fluid (CSF) of patients with PD shows reduced levels of progesterone metabolites, such as allopregnanolone [31]. In addition, peripheral blood mononuclear cells exhibit decreased levels of enzymes involved in progesterone metabolism, including 5α-reductase type 1, and reduced expression of this enzyme has also been observed in substantia nigra neurons, resulting in lower levels of allopregnanolone [31].

Allopregnanolone and its precursor, 5α-dihydroprogesterone (5α-DHP), play critical roles in protecting neurons against NMDA- and kainic acid–induced excitotoxicity, reducing apoptosis, and promoting normal myelination and proliferation of neural progenitor cells [31]. These compounds therefore have important neuroprotective functions, and their reduced synthesis or metabolism may contribute to neuronal degeneration in the substantia nigra [31].

In addition, the neuroactive steroid progesterone modulates the main striatal neurotransmitter systems involved in the pathophysiology of the PD [35].

Despite these potential neuroprotective effects, our meta-analysis showed that estrogen-only HT was not associated with either a reduced or increased risk of PD, whereas combined therapy was associated with an increased risk (pooled estimate 1.40).

We hypothesize that, during menopause, multiple mechanisms may converge to increase the risk of PD even in the context of HT. These mechanisms can be broadly summarized into three key domains:

### Time of initiation

Following the publication of the Women’s Health Initiative (WHI) results [37], the “critical window of opportunity” hypothesis was proposed to explain the effects of estrogen therapy. According to this hypothesis, estrogen therapy is beneficial when initiated close to menopause or the cessation of ovarian function, whereas initiation outside this time window may be ineffective or even detrimental [38].

One proposed mechanism is that prolonged deprivation of ovarian hormones leads to a reduction in the density of estrogen receptors (ER), particularly ERα, in neuronal membranes [38]. An animal study by Bohacek and Daniel (2009) [39] demonstrated that long-term ovarian hormone deprivation results in increased degradation of ERα via the ubiquitin–proteasome system. The authors hypothesized that the absence of estrogen during a critical period following ovarian failure leads to a persistent loss of ERα, which may not be reversed by delayed estrogen administration [39].

A similar concept has been proposed for progesterone, with evidence suggesting that aging affects the expression, binding activity, and distribution of progesterone receptors in the brain [36]. Although much of this evidence derives from hippocampal models, and dopaminergic neurons in the substantia nigra predominantly express ERβ [40], estrogen deprivation and aging are known to alter ERβ expression and sensitivity in a region-specific manner [41].

Therefore, it is plausible that a similar critical window mechanism may also operate in the substantia nigra during later stages of menopause.

### Order of hormone use

Although both estrogen and progesterone exhibit neuroprotective properties, evidence suggests that their effects are not necessarily synergistic and may be attenuated when administered simultaneously [34].

In dopaminergic neurons from animal models, progesterone and its main metabolite, allopregnanolone, have been shown to increase dopaminergic tone by stimulating dopamine release. However, this effect appears to depend on prior exposure of these neurons to estrogen [34].

These findings suggest that the sequence of hormonal exposure may be critical for achieving optimal neuroprotective effects. This mechanism may partially explain why combined HT, when administered concurrently, does not reproduce the potential neuroprotective effects observed with estrogen alone.

### Type of molecules used in HT

Several studies suggest that the type of progesterone used in HT is a critical determinant of its neurological effects. Natural (bioidentical) progesterone (P4) has been associated with more pronounced neuroprotective properties, including reduction of oxidative stress and neuronal cell death [42]. In addition, the combination of estrogen with natural progesterone has been associated with a greater reduction in the risk of neurodegenerative diseases [10].

In contrast, the synthetic progestin medroxyprogesterone acetate (MPA), one of the most commonly used progestins in HT [37, 43, 44], has been associated with reduced neuroprotective effects. Experimental evidence suggests that MPA may impair the conversion of progesterone into allopregnanolone [34], leading to decreased myelination, reduced efficacy of estrogen-mediated neuroprotection [10, 34, 43, 45], increased glutamate-mediated excitotoxicity [45] and impaired mitochondrial function [43]. These alterations may further contribute to metabolic dysregulation affecting synaptic function and neuronal signaling [43]. This distinction between natural and synthetic progestins may partly explain the increased risk observed with combined HT in our meta-analysis.

From a clinical perspective, these findings do not support the use of combined HT for neuroprotection and highlight the need for individualized risk-benefit assessment when prescribing HT in postmenopausal women. Furthermore, it may reflect differential modulation of dopaminergic neurodegeneration by specific hormone formulation.

This study has several notable strengths. It was conducted in accordance with PRISMA guidelines, employed a comprehensive search strategy, and included cohort and case–control studies. The inclusion of formulation-specific analyses, a large combined sample size, and studies with long follow-up periods enhances the robustness and generalizability of the findings.

However, some limitations should be acknowledged. First, all included studies were observational, which precludes causal inference. Second, moderate heterogeneity was observed, likely reflecting differences in study design, population characteristics, and definitions of HT exposure. Third, diagnostic criteria for PD varied across studies, with many relying on clinical judgment without standardized criteria. Fourth, some studies contributed more than one formulation-specific estimate, which may introduce within-study dependence in subgroup analyses. Fifth, the relatively small number of studies in subgroup analyses may have limited statistical power to detect interaction effects. Finally, residual confounding cannot be excluded, particularly regarding timing, duration, and formulation of HT.

In addition, residual confounding cannot be excluded. In particular, confounding by indication may have influenced the observed associations, as women prescribed hormone therapy may differ systematically from non-users in terms of baseline health status, menopausal symptoms, and healthcare utilization.

Furthermore, timing bias related to the initiation of HT (early versus late postmenopausal period) was not consistently reported across studies and may represent an important source of heterogeneity, especially in light of the “critical window” hypothesis.

Finally, formulation heterogeneity should be considered, as different progestin compounds (e.g., medroxyprogesterone acetate versus natural progesterone) may exert distinct biological effects, which could not be adequately explored in the present analysis due to limited data availability.

In conclusion, this systematic review and meta-analysis indicates that combined menopausal HT was associated with an increased risk of PD, whereas no significant association was observed for estrogen-only therapy. These findings raise important considerations regarding the neurological safety of specific HT formulations and should be considered hypothesis-generating.R.

The underlying mechanisms remain incompletely understood, particularly regarding the timing of therapy initiation and the interaction between estrogen and progestins. Further well-designed studies are needed to clarify these relationships and to guide safer and more individualized therapeutic strategies in menopausal care.

## Electronic Supplementary Material

Below is the link to the electronic supplementary material.

Supplementary material 1 (20.0 KB)

Supplementary material 2 (33.8 KB)

Supplementary material 3 (23.2 KB)

Supplementary material 4 (857 KB)
