# Supplementary material for: Differential effects of hormone therapy formulations on Parkinson’s disease risk: a systematic review and meta-analysis
Source: Neurol Sci. 2026 Jul 22;47(8):651. doi: 10.1007/s10072-026-09243-6 (PMC13388350; doi:10.1007/s10072-026-09243-6)
Supplement: Supplementary file 1 [file 10072_2026_9243_MOESM1_ESM.odt]

Full search strategy for each database

PubMed

("Parkinson Disease"[Mesh] OR "Parkinson's disease" OR parkinson*) AND ("Estrogens"[Mesh] OR estrogen* OR oestrogen* OR estradiol OR "Hormone Replacement Therapy"[Mesh] OR "Menopausal Hormone Therapy" OR "Sex Hormones"[Mesh] OR "Estrogen Replacement Therapy”) AND (menopause OR menopausal OR "Age at Menopause”[Mesh])

EMBASE

('parkinson disease'/exp OR 'parkinson disease' OR parkinson*) AND ('estrogen'/exp OR estrogen* OR oestrogen* OR estradiol OR 'hormone replacement therapy'/exp OR 'menopausal hormone therapy’) AND ('menopause'/exp OR menopause OR menopausal OR ‘early menopause')
