# Supplementary material for: Differential effects of hormone therapy formulations on Parkinson’s disease risk: a systematic review and meta-analysis
Source: Neurol Sci. 2026 Jul 22;47(8):651. doi: 10.1007/s10072-026-09243-6 (PMC13388350; doi:10.1007/s10072-026-09243-6)
Supplement: Supplementary file 2 [file 10072_2026_9243_MOESM2_ESM.odt]

| # Supplementary file 2 - PD diagnosis criteria for each study |
| --- |
| # Study (year) | # PD criteria |
| Yuk (2023) | # ICD |
| Ascherio (2003) | # Treating neurologist judgment |
| Popat (2005) | Presence of at least two of the following signs: bradykinesia, cogwheel rigidity, resting tremor, and postural reflex impairment, at least one of which must be resting tremor or bradykinesia, plus no suggestion of a cause for another parkinsonian syndrome, no atypical features; and two of the following features: asymmetric onset, symptomatic improvement after treatment with L-dopa, or a progressive disease course. |
| Rugbjerg (2013) | # ICD |
| Nicoletti (2011) | Gelb |
| Liu (2014) | Treating neurologist judgment or evidence of two or more cardinal signs of PD (with one being rest tremor or bradykinesia), a progressive course, responsiveness to dopaminergic treatments, and absence of features that suggested an alternative diagnosis. |
| Benedetti (2001) | presence of parkinsonism with all three of: (1) no other cause; (2) no documentation of unresponsiveness to levodopa at doses of at least 1 g/day in combination with carbidopa (applicable only to patients who were treated); (3) no prominent or early (within 1 year of onset) signs of more extensive nervous system involvement not explained otherwise. |
| Simon (2009) | # Treating neurologist judgment or evidence at a neurological examination of at least two of the three cardinal signs (rest tremor, rigidity, and bradykinesia) in the absence of clinical features suggesting other diagnoses or nonresponsiveness to levodopa treatment. |
| Currie (2004) | Presence of at least 2 of the 3 cardinal features of PD and no atypical features. |
| Pesce (2023) | 1- Definite PD: Presence of at least 2 out of 4 cardinal signs (rest tremor, rigidity, bradykinesia, impaired postural reflexes), with a positive and significant response to treatment at a sufficient dose, without prominent or early signs of more extensive nervous system involvement and drug-induced parkinsonism. 2- Probable PD: When the same criteria were fulfilled but there was insufficient information regarding treatment response. 3- Possible PD: when the clinical description was suggestive of PD, but there was insufficient information in the medical records to assign a definite or probable PD diagnosis. |
| Gatto (2014) | Gelb and UK brain bank |
| Marder (1998) | # Presence of bradykinesia and at least one of the following: muscular rigidity, resting tremor, or postural instability. Additional criteria also included the presence of at least three of the following: a history of unilateral onset, persistence of the asymmetry of signs or symptoms, a history of improvement on levodopa, a progressive course, or a history of levodopa-induced chorea. |
| Ragonese (2004) | Presence of two of four cardinal signs (tremor at rest, rigidity, bradykinesia, and impaired postural reflexes) plus the following requirements: 1) unilateral onset, progressive course, unchanging asymmetry of signs (signs of parkinsonism persistently asymmetric at follow-up evaluation); and 2) good responsiveness to antiparkinsonian drugs (L-dopa or dopaminoagonists) in treated persons. |
| Greene (2014) | Not infofrmed |
| UK - United Kingdom; ICD - International classification of disease; PD - Parkinson disease; SD - Standard deviation; USA - United States of America; NA - Not applicable; NI - Not informed |
