# Supplementary material for: Differential effects of hormone therapy formulations on Parkinson’s disease risk: a systematic review and meta-analysis
Source: Neurol Sci. 2026 Jul 22;47(8):651. doi: 10.1007/s10072-026-09243-6 (PMC13388350; doi:10.1007/s10072-026-09243-6)
Supplement: Supplementary file 3 [file 10072_2026_9243_MOESM3_ESM.odt]

| # Supplementary file 3 - Newcastle-Ottawa Scale (NOS) of the included studies |
| --- |
| # Study (year) | # Design | # Selection | # Compatibility | # Outcome/Exposition | # Total score (classification) |
| Marder (1998) [18] | # Cohort | ✭✭✭ | # ✭ | # ✭✭ | # 6 (Moderate risk) |
| Benedetti (2001) [19] | # Case control | # ✭✭✭✭ | # ✭ | # ✭✭✭ | # 8 (Low risk) |
| Ascherio (2003) [20] | # Cohort | # ✭✭✭✭ | # ✭✭ | # ✭✭✭ | # 9 (Low risk) |
| Currie (2004) [21] | # Case control | ✭✭✭ | # ✭ | # ✭✭ | # 6 (Moderate risk) |
| Ragonese (2004) [6] | # Case control | # ✭✭✭✭ | # ✭✭ | # ✭✭ | # 8 (Low risk) |
| Popat (2005) [22] | # Case control | # ✭✭✭✭ | # ✭✭ | # ✭✭ | # 8 (Low risk) |
| Simon (2009) [23] | # Cohort | # ✭✭✭✭ | # ✭✭ | # ✭✭✭ | # 9 (Low risk) |
| Nicoletti (2011) [24] | # Case control | # ✭✭ | # ✭✭ | # ✭✭ | # 6 (Moderate risk) |
| Rugbjerg (2013) [25] | # Cohort | # ✭✭✭✭ | # ✭✭ | # ✭✭✭ | # 9 (Low risk) |
| Liu (2014) [26] | # Cohort | # ✭✭✭✭ | # ✭✭ | # ✭✭✭ | # 9 (Low risk) |
| Gatto (2014) [27] | # Case control | # ✭✭✭✭ | # ✭✭ | # ✭✭✭ | # 9 (Low risk) |
| Greene (2014) [28] | # Case control | # ✭✭✭✭ | # ✭✭ | # ✭✭✭ | # 9 (Low risk) |
| Yuk (2023) [29] | # Cohort | # ✭✭✭✭ | # ✭✭ | # ✭✭✭ | # 9 (Low risk) |
| Pesce (2023) [30] | # Cohort | # ✭✭✭✭ | # ✭✭ | # ✭✭✭ | # 9 (Low risk) |
| UK - United Kingdom; ICD - International classification of disease; PD - Parkinson disease; SD - Standard deviation; USA - United States of America; NA - Not applicable; NI - Not informed |
