# Supplementary material for: Differential effects of hormone therapy formulations on Parkinson’s disease risk: a systematic review and meta-analysis
Source: Neurol Sci. 2026 Jul 22;47(8):651. doi: 10.1007/s10072-026-09243-6 (PMC13388350; doi:10.1007/s10072-026-09243-6)
Supplement: Supplementary file 4 [file 10072_2026_9243_MOESM4_ESM.odt]

Agruparleave-one-out 14.pngLeave-one-out analysis of overall hormone therapy

Funnel plot 14.png
